# Hydrometeorology and flood pulse dynamics drive diarrheal disease outbreaks and increase vulnerability to climate change in surface-water-dependent populations: A retrospective analysis

**DOI:** 10.1371/journal.pmed.1002688

**Published:** 2018-11-08

**Authors:** Kathleen A. Alexander, Alexandra K. Heaney, Jeffrey Shaman

**Affiliations:** 1 Department of Fish and Wildlife Conservation, Virginia Tech, Blacksburg, Virginia, United States of America; 2 Chobe Research Institute, Centre for Conservation of African Resources: Animals, Communities, and Land Use, Kasane, Botswana; 3 Department of Environmental Health Sciences, Mailman School of Public Health, Columbia University, New York, New York, United States of America; University of Wisconsin, Madison, UNITED STATES

## Abstract

**Background:**

The impacts of climate change on surface water, waterborne disease, and human health remain a growing area of concern, particularly in Africa, where diarrheal disease is one of the most important health threats to children under 5 years of age. Little is known about the role of surface water and annual flood dynamics (flood pulse) on waterborne disease and human health nor about the expected impact of climate change on surface-water-dependent populations.

**Methods and findings:**

Using the Chobe River in northern Botswana, a flood pulse river—floodplain system, we applied multimodel inference approaches assessing the influence of river height, water quality (bimonthly counts of *Escherichia coli* and total suspended solids [TSS], 2011–2017), and meteorological variability on weekly diarrheal case reports among children under 5 presenting to health facilities (*n* = 10 health facilities, January 2007–June 2017). We assessed diarrheal cases by clinical characteristics and season across age groups using monthly outpatient data (January 1998–June 2017). A strong seasonal pattern was identified, with 2 outbreaks occurring regularly in the wet and dry seasons. The timing of outbreaks diverged from that at the level of the country, where surface water is largely absent. Across age groups, the number of diarrheal cases was greater, on average, during the dry season. Demographic and clinical characteristics varied by season, underscoring the importance of environmental drivers. In the wet season, rainfall (8-week lag) had a significant influence on under-5 diarrhea, with a 10-mm increase in rainfall associated with an estimated 6.5% rise in the number of cases. Rainfall, minimum temperature, and river height were predictive of *E*. *coli* concentration, and increases in *E*. *coli* in the river were positively associated with diarrheal cases. In the dry season, river height (1-week lag) and maximum temperature (1- and 4-week lag) were significantly associated with diarrheal cases. During this period, a 1-meter drop in river height corresponded to an estimated 16.7% and 16.1% increase in reported diarrhea with a 1- and 4-week lag, respectively. In this region, as floodwaters receded from the surrounding floodplains, TSS levels increased and were positively associated with diarrheal cases (0- and 3-week lag). Populations living in this region utilized improved water sources, suggesting that hydrological variability and rapid water quality shifts in surface waters may compromise water treatment processes. Limitations include the potential influence of health beliefs and health seeking behaviors on data obtained through passive surveillance.

**Conclusions:**

In flood pulse river—floodplain systems, hydrology and water quality dynamics can be highly variable, potentially impacting conventional water treatment facilities and the production of safe drinking water. In Southern Africa, climate change is predicted to intensify hydrological variability and the frequency of extreme weather events, amplifying the public health threat of waterborne disease in surface-water-dependent populations. Water sector development should be prioritized with urgency, incorporating technologies that are robust to local environmental conditions and expected climate-driven impacts. In populations with high HIV burdens, expansion of diarrheal disease surveillance and intervention strategies may also be needed. As annual flood pulse processes are predominantly influenced by climate controls in distant regions, country-level data may be inadequate to refine predictions of climate—health interactions in these systems.

## Abstract 2

Kathleen Alexander and colleagues determine the effects of river flood height, water quality and amount of suspended solids in river water in Botswana in response to climate change and weather variability and the resultant cases of diarrhoea in children under 5.

## Author summary

**Why was this study done?:**

- Diarrheal disease remains a persistent global health threat for children under 5 years of age, particularly in Africa.
- In river—floodplain systems, annual floods or flood pulses can occur, with extreme flooding and drought a common threat to local populations. We do not know how surface water dynamics influence diarrheal disease and human health or how climate change will affect populations dependent on surface water resources in these regions.

**What did the researchers do and find?:**

- We conducted a study on the Chobe River floodplain system in northern Botswana, a system that floods annually and is the source of drinking water for 8 villages included in the study.
- We evaluated diarrheal outbreak patterns in children under 5 years of age (weekly, 2007–2017) and individuals of all ages (monthly, 1998–2017) reporting diarrheal disease at government health facilities (*n* = 10).
- Using multimodel inference approaches, we assessed how meteorology, river height, and water quality characteristics (2011–2017) were associated with reports of diarrheal disease in children under 5 years of age.
- Across individuals of all ages, diarrheal outbreaks occurred regularly in the wet and dry seasons.
- Season had an important influence on the type of diarrhea and age group of the patient.
- Increases in diarrheal case reports were closely tied to meteorology, flood recession, and decreases in surface water quality, with the highest number of cases occurring in the dry season.

**What do these findings mean?:**

- In flood pulse river—floodplain systems, rapid changes in surface water quality may influence the ability of water treatment plants to provide safe drinking water.
- Climate change is expected to increase hydrological variability, further influencing the vulnerability of populations in this region.
- There is an urgent need to strengthen water infrastructure to address current and future environmental impacts that can influence the production of safe water.
- In populations with high HIV infection levels, public health focus may need to be extended to include other at-risk groups beyond children under five, such as HIV-infected individuals and others that may also be immunocompromised.
- As distant weather patterns influence local flood dynamics, use of country-level data may not be adequate to predict climate impacts on human health in these types of systems.

## Introduction

Across land types, the flow of water (ground water, surface water, and rainfall) has been demonstrated to influence infectious disease transmission dynamics across scales, from microbial to host levels [1–4]. Degradation of freshwater ecosystems and declines in water quality in surface water represent a growing public health threat across Africa [5]. Worldwide, degraded water quality, waterborne disease, and infectious diarrhea remain prominent and persistent concerns that are expected to worsen under future climate change projections [6,7]. In 2015, diarrheal disease was estimated to cause 1.31 million deaths per year [8]. Of these, nearly half a million deaths occurred in children under 5 years of age, with the greatest burden of disease focused in sub-Saharan Africa and South Asia [9,10]. Diarrheal disease in children can have lasting population effects, such as stunting and cognitive deficiencies [11]. Chronic oral exposure to fecal contaminates is also linked to environmental enteropathy, a subclinical disorder marked by chronic gut inflammation, small bowel structural change, decreased gut permeability, and gut immune dysfunction, which can influence oral vaccine efficacy [12]. The societal impact of diarrheal disease in children can be lasting, underscoring the need to advance our understanding of environmental couplings to develop effective public health strategies that will not only reduce current disease burdens but minimize future climate change impacts.

Floods are identified as the most pervasive hydrometeorological hazard (reviewed in [13]), are expected to increase in frequency under climate change [14], and are linked strongly with the occurrence of diarrheal disease outbreaks in affected populations [15–18]. However, flood and flow regimes can vary significantly by waterway, and range from large rivers in temperate regions with more regular flow to dryland river—floodplain systems in arid and semiarid regions where highly variable flow occurs annually (flood pulse), with extreme flooding as well as droughts a common outcome [19]. In flood pulse systems, there is a predictable advance of floodwaters onto surrounding floodplains and retraction back into the river channel with flood recession, linking aquatic and terrestrial landscapes and microbial communities. These annual flood dynamics are highly variable and can change dramatically in flow volume, duration, frequency, and timing [20,21]. These systems tend to be in a state of dynamic equilibrium, with system behavior and biota characterized by flood dynamics and the geomorphology of the river—floodplain system [21]. These interacting hydrological, geomorphological, and sedimentary processes are fundamental determinants of microbial dynamics, water quality, and waterborne pathogen exposure in humans and animals in flood-prone areas but have not yet been adequately characterized, hampering our ability to address current and climate-mediated population vulnerabilities.

Botswana provides an ideal model system to isolate the role of surface water and flood dynamics on climate—diarrheal disease couplings. Botswana is an arid to semiarid country in Southern Africa with only 3 sources of permanent surface water throughout the country. Rainfall is limited and only occurs during a defined wet season, with little or no rainfall during the dry season. At the national level, diarrheal disease remains a persistent health threat to children and adults alike [22]. Using this study system and a unique dataset, we evaluated the influence of meteorology, surface water quality, and flood pulse dynamics on diarrheal disease and discuss the implications for public health and climate preparedness needs in systems where populations are dependent on surface water resources.

## Methods

To analyze the relationships between variables, we used multimodel inference, which allowed quantification of model uncertainty among different candidate models and unconditional inference across those different models [23]. These methods permit inference of important predictor variables, estimation of the average effects of predictor variables on an outcome, and generation of multimodel weighted averaged predictions of that outcome.

### Study site

**Fig 1:**
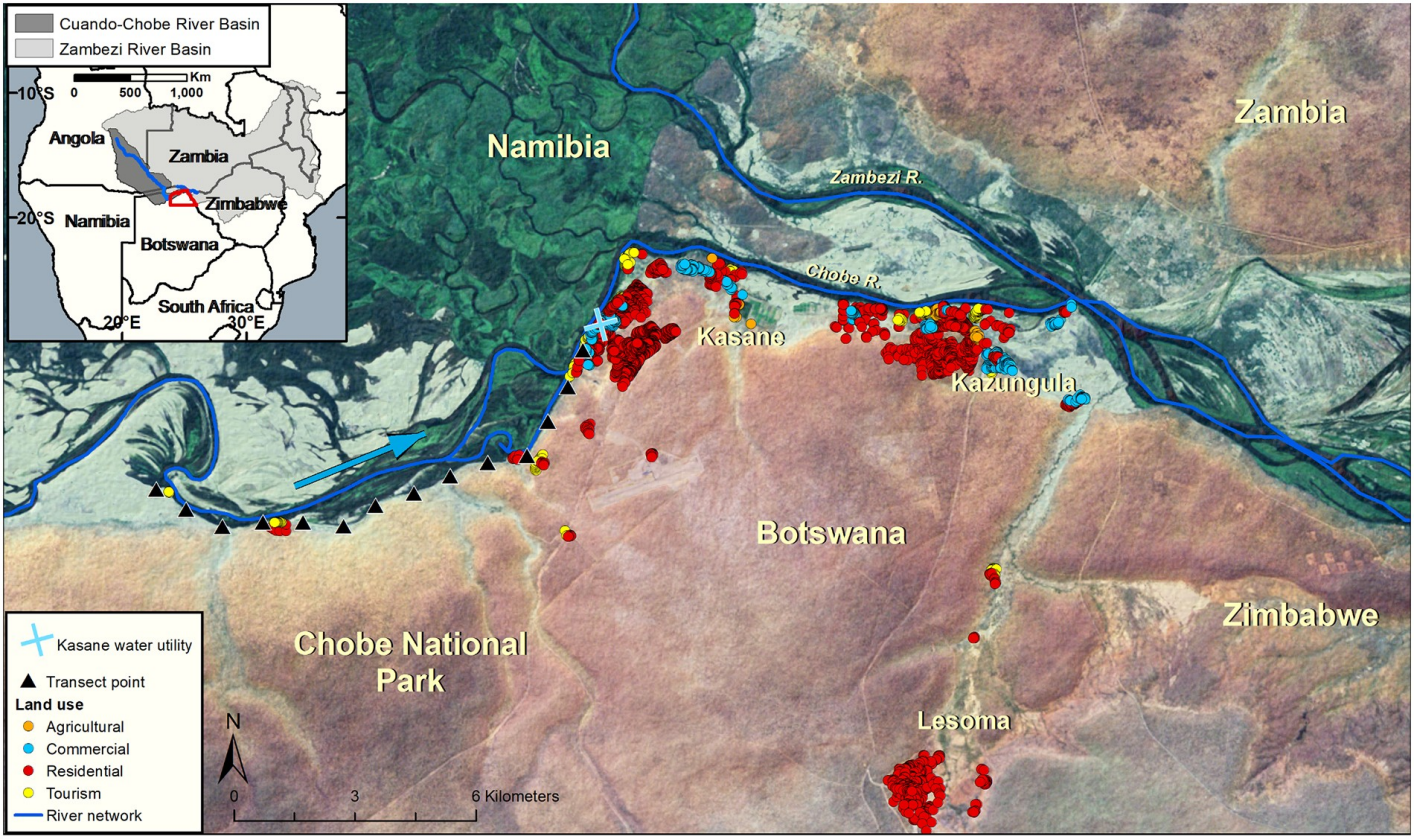
The study was conducted in northern Botswana in Southern Africa. The Chobe River (blue line) is a transboundary waterway and 1 of only 3 perennial sources of water within Botswana, with water flow (light blue arrow) moving from the national park towards the urban areas, where the water treatment facility is located (blue X). Water quality samples were collected biweekly at established transect points (black triangles). Surface waters are abstracted to produce drinking water through centralized water treatment facilities and distributed to the population. Circles represent buildings color coded by the type of infrastructure (agricultural, commercial, residential, or tourism related; see legend). In this system, annual floods are driven by distant and regional wet season precipitation, with the largest input arising from floodwaters that occur in association with tropical rains in the upper watersheds of the Zambian and Angolan Highlands (inset, northern aspect of the Cuando-Chobe River Basin). The peak of the flood pulse arrives in the Chobe River at the end of the wet season/beginning of the dry season, after traversing more than 1,000 km from the highland areas.

Botswana is a semiarid, landlocked country in Southern Africa. The country has a subtropical climate with annual wet (November—March) and dry (April—October) seasons. Intra- and inter-annual precipitation variability is high, resulting in frequent droughts and flooding. This study focuses on Chobe District, located in the northeastern part of Botswana (Fig 1). The Chobe River floodplain system, which is the primary source of water for the district, floods annually (flood pulse), with the peak flood height occurring in the late wet season/early dry season (April). The district contains 1 primary hospital, 3 private clinics, and 12 government health clinics that serve a total population of approximately 25,000 people (Central Statistics Office of Botswana, 2011). Medical services are provided by the government, with patients paying a nominal fee for health services. Chobe District is also home to the Chobe National Park, which provides an important habitat for the largest elephant population in Africa, as well as an abundance of other wildlife species (Fig 1).

The Chobe River supplies all domestic water needs for the study villages (*n* = 8), providing a unique environment to assess the impact of surface water dynamics on the health of dependent populations. Water is pumped directly from the river into a conventional water treatment plant and then distributed through direct reticulation to households (private outdoor and indoor taps) or to public taps [22]. River water traverses a protected area before it reaches the water treatment plant (Fig 1). Turbidity and pH are measured manually; values are used to determine the amount of coagulant that is automatically deployed over time in the water treatment process. A second fully automated plant was deployed in 2014, serving 2 out of the 8 study villages. Since 2016, frequent—daily and sometimes multiple daily—water purges occur in the second system, which continues to date. The majority of households have access to improved sanitation, with only 14% of households reporting lack of access in a recent survey of 3 of the study villages, most frequently due to pit latrines being at capacity [24]. At the country level, diarrheal disease outbreaks occur in a bimodal, cyclical pattern peaking during March and October in the wet and dry seasons, respectively [25].

### Time series data

#### Hydrometeorological data

Data period, type, and spatial resolution are provided in Table 1. Meteorological data were acquired from the Department of Meteorological Services under the Ministry of Environment, Natural Resources Conservation and Tourism. Measurements from all sites were averaged to produce daily estimates of temperature and rainfall in Chobe District. The Department of Water Affairs provided daily measurements of the Chobe River height. All hydrometeorological variables were aggregated to a weekly resolution and span from January 2007 to June 2017.

**Table 1:** Data type and resolution used in the analysis.

Category	Variable measured	Temporal scale	Number of measurements	Temporal resolution	Spatial scale
**Disease data**	Under-5 diarrhea cases	Jan. 2007–Jun. 2017	10 health facilities	Weekly total	Chobe District
	Outpatient diarrhea cases	Jan. 1998–Jun. 2017	10 health facilities	Monthly total	Chobe District
**Hydrometeorological data**	Maximum temperature	Jan. 2007–Jun. 2017	1 station	Weekly average	Chobe District
	Minimum temperature	Jan. 2007–Jun. 2017	1 station	Weekly average	Chobe District
	Rainfall	Jan. 2007–Jun. 2017	2 stations	Weekly sum	Chobe District
	River height	Jan. 2007–Jun. 2017	1 station	Weekly average	Chobe River
**Water quality**	*E*. *coli*	Jul. 2011–Jun. 2014, Jul. 2015–Jul. 2017	14 transect points	Biweekly sampling, averaged over transects	27.5 km of Chobe River
	Total suspended solids	Jul. 2011–Jun. 2014, Jul. 2015–Jul. 2017	14 transect points	Biweekly sampling, averaged over transects	27.5 km of Chobe River

#### Water quality assessments

Water grab samples were collected bimonthly from established transect sites (*n* = 14 sampling points, July 2011–July 2017; Fig 1) located at 1-km intervals along the Chobe River above the 2 water intake stations for the community. Land use along this reach of the river consisted of protected area and urban land use. Water quality assessments were conducted to estimate in-river concentrations of *E*. *coli* and total suspended solids (TSS), as previously described [26].

#### Diarrheal case reports

Diarrheal case reports were obtained for the study from 10 health facilities (9 government health clinics and 1 primary hospital) through 2 passive surveillance systems operated under the Botswana Ministry of Health (MoH). Under-5 diarrhea case data were acquired from the Integrated Disease Surveillance and Response (IDSR) program (January 2007–June 2017), which collates weekly numbers of under-5 diarrhea cases presenting to district health facilities (Table 1). Total monthly outpatient cases of diarrhea (all ages) were also obtained from the MoH for January 1998–June 2017. These case reports were extracted from the same 10 health facilities, stratified by diarrhea type, age (<1, 1–4, 5+ years), and sex of the patient. Outpatient data include the IDSR weekly data (a subset) as all patients must access the health system through outpatient services. These data are useful as they provide information on all patients by age over and above those less than 5 years. Outpatient data were included to provide a more complete demographic and clinical picture of diarrheal disease in the district. A diarrhea case was defined as the occurrence of at least 3 loose stools in a 24-hour period within the 4 days preceding the health facility visit. The diarrhea type was characterized by the attending physician or nurse as diarrhea with no dehydration, diarrhea with some dehydration, diarrhea with severe dehydration, or bloody diarrhea. Each selected study village had a clinic, with the largest town, Kasane, having 2 clinics and a primary hospital (*n* = 3). For both the IDSR and outpatient datasets, data from the clinic in the village of Pandamatenga were excluded because residents in this village obtained water from boreholes and were expected to have variable exposure to surface water resources from other villages and towns in the district. Demographic statistical analyses were conducted using chi-squared tests for differences in proportions between groups.

Diagnoses were categorized according to the International Classification of Diseases–10th Revision (ICD-10 [27]). Case data represent summary clinical diagnoses of attending physicians or nurses in government health facilities and were not associated with any clinical diagnostic information.

### Correcting for missing under-5 diarrhea data

In the IDSR record, missing data existed for each of the 10 reporting health facilities. Weeks with no reports (i.e., all 10 health facilities not reporting) were not included in the analysis. We used 2 approaches to correct for the missing reports for weeks where data gaps existed (i.e., data from fewer than 10 health facilities). In the first approach, we took the total number of cases reported in a given week and divided this by the number of health facilities reporting that week. This method provides a weekly estimate of the total under-5 diarrhea cases per health facility reporting but does not account for differences in patient volume. For the second method, observations were first standardized as follows:

$$x_{t,i}^{s}=\frac{x_{t,i}-\mu_{i}}{\sigma_{i}}$$

where $x_{t,i}^{s}$ is the standardized observation for week *t* and health facility *i*, *x*_*t*,*i*_ is the raw number of observations for week *t* and health facility *i*, μ_*i*_ is the mean number of observations for health facility *i*, and σ_*i*_ is standard deviation of the number of observations for health facility *i*. For each week, those standardized numbers of observations were averaged across reporting health facilities to create a district-wide standardized estimate, $x_{t}^{s}$, i.e.,

$$x_{t}^{s}=\sum_{j=1}^{n}\frac{x_{t,i}^{s}}{n}$$

where *n* is the number of clinics reporting in a given week. This standardized weekly under-5 diarrhea estimate, $x_{t}^{s}$, represents the average deviation from the mean number of cases across reporting clinics. Analyses were performed using both correction approaches, and results were consistent with both data forms. Findings using weekly under-5 diarrhea data divided by the number of reporting clinics are presented here; findings using standardized under-5 diarrhea data are presented in S3 Table and S3–S5 Figs. Overall, variable importance and effect size were consistent irrespective of which data correction method was used.

### Environmental predictors of under-5 diarrhea and water quality: Multimodel inference

Three separate analyses were performed to determine the associations between environmental variables and weekly under-5 diarrhea cases, *E*. *coli*, and TSS. The analyses differed only in the outcome of interest and the basic structure of the models. Since water quality variables were measured over a shorter time period, the models predicting *E*. *coli* and TSS included fewer observations (*n* = 42 [wet season], *n* = 54 [dry season]) than models without water quality variables (*n* = 164 [wet season], *n* = 236 [dry season]). A priori, we believed that different environmental drivers were important for the dry and wet season outbreaks of diarrhea in children under 5. Hence, analyses were performed separately for the wet and dry seasons.

Multimodel inference was carried out using groups of candidate regression models, each developed with the same structure but different predictor variables. Under-5 diarrhea observations, which are overdispersed count data, were modeled using negative binomial regression. *E*. *coli* and TSS concentration data were Gaussian in structure and were modeled using ordinary least squares regression. Models for under-5 diarrhea were developed using all combinations of 12 different environmental variables: minimum temperature (lagged 1, 4, and 8 weeks), maximum temperature (lagged 1, 4, and 8 weeks), rainfall (lagged 1, 4, and 8 weeks), and Chobe River height (lagged 1, 4, and 8 weeks). Models for *E*. *coli* or TSS included the same environmental variables lagged 0 and 4 weeks. These lag times were chosen to represent the acute (0/1 week), short-term (4 week), and long-term (8 week) effects of environmental variability. All regressions included a dummy variable for year and an autoregressive term (diarrhea incidence lagged 1 week) as predictor variables. This resulted in 4,096 candidate models of under-5 diarrhea for both the wet and dry season, and 256 candidate models for both *E*. *coli* and TSS for each season. In summary, within a multimodel ensemble, every model had the same regression structure (either Gaussian or negative binomial) and included a dummy variable for year and an autoregressive term. The only differences between the models were the environmental variables included as predictors.

#### Multimodel inference methods

Five different subsets of the candidate models were created by (1) retaining all models, (2) selecting models with all coefficients statistically significant at *p* < 0.20, (3) selecting models with all coefficients statistically significant at *p* < 0.10, (4) selecting models with all coefficients statistically significant at *p* < 0.05, and (5) selecting only the model with the lowest Akaike information criterion, corrected for the small sample size relative to the number of parameters used in each model (AICc). The Akaike weight of each model within the subset was then calculated. Akaike weights provide a relative likelihood that a specific model is the best model in a given subset [23]. “Top” model sets were then obtained by selecting the smallest combination of models with Akaike weights summing to 0.95.

These top model sets were used to calculate averaged variable coefficients and relative variable importance. The average coefficient for variable *X* was calculated by taking a weighted average of the coefficient estimates for variable *X* from each subset model. Each coefficient estimate was weighted by the Akaike weight of its respective model. Relative variable importance is a measure of how often a predictor variable is included in the best performing models. The relative importance of variable *X* was calculated by adding the Akaike weights of all models that included variable *X* as a predictor. These importance measurements do not add up to 1 because models can include multiple environmental predictors.

#### Multimodel averaged predictions

Multimodel averaged predictions are simply the Akaike weighted average of the subset constituent model predictions. Pseudo *R*-squared was calculated for the averaged model, top model, and null model by squaring the correlation between the averaged predictions and observations of weekly diarrhea. In addition, leave-one-out (LOO) cross-validation was performed by omitting 1 year of data from model fitting and then generating and evaluating model averaged predictions for the missing year. Because our models include a dummy variable for year, which cannot be estimated for the omitted year, correlation rather than root mean square error (RMSE) was used to evaluate model predictive performance.

### Analysis of water quality and under-5 diarrhea

Negative binomial regressions were used to evaluate the relationships between Chobe River water quality and under-5 diarrhea case reports. Multiple models were run with *E*. *coli* or TSS lagged from 0 to 8 weeks as the predictor variable and under-5 diarrhea cases as the outcome.

Each model, which included an autoregressive term (under-5 diarrhea incidence lagged 1 week) and a dummy variable for year, contained only 1 lagged water quality predictor variable. Each model included data from weeks with complete observations for all predictors.

### Study permissions and approvals

This study was conducted under permit from the Ministry of Environment, Natural Resources Conservation and Tourism (EWT8/36/4) and the MoH (HPSME:13/18/1 Vol. X [878]). Approval was also obtained from the Virginia Tech Institutional Review Board (#11–573).

## Results

**Fig 2:**
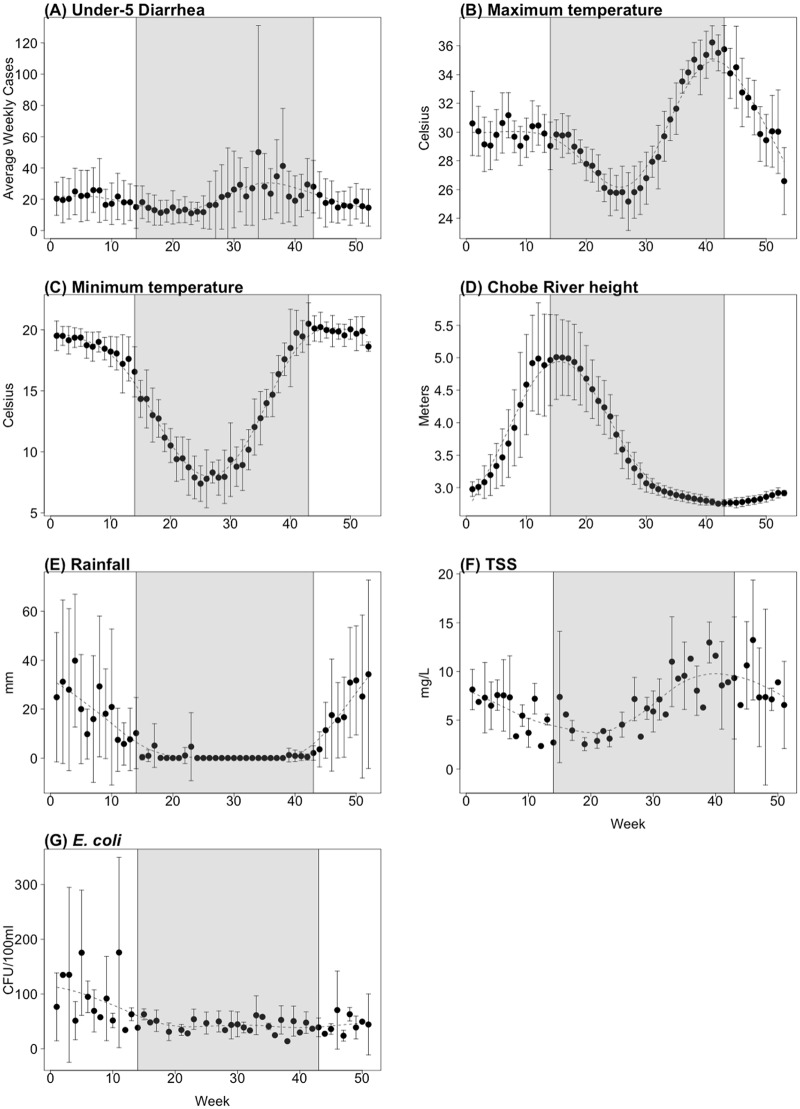
Seasonal plots of all variables. All plots represent the average weekly values across all years of data. The shaded portion represents the dry season, and the dashed black line is a cubic spline fit through the data to show seasonal trend. Average weekly number of under-5 diarrheal cases reported for 2007–2017 is shown in (A); (B—E) provide the average weekly values for environmental variables over the study period from January 2007 to June 2017 (full time series data are provided in S1 Fig), and (F) and (G) are average biweekly water quality measurements in the Chobe River for 2011–2017. CFU, colony-forming units; TSS, total suspended solids.

IDSR weekly cases of under-5 diarrhea in Chobe District exhibited strong seasonal dynamics over the decade of observation (2007–2017), with 2 outbreaks occurring regularly each year (Fig 2). The first outbreak period occurred on average in late January during the wet season, and the second, in August during the dry season. The diarrheal attack rate was greater, on average, for dry season outbreaks (average number of cases = 615, SD = 333) than for wet season outbreaks (average number of cases = 407, SD = 232). The number of outpatient diarrhea cases reported per month (all ages) for the same area exhibited a similar seasonal pattern. As with under-5 diarrhea, the all-age outpatient attack rate tended to be higher on average in the dry season (average = 725, SD = 381) than in the wet season (average = 466, SD = 226).

### Demographic and clinical characteristics of diarrheal disease

Outpatient (monthly, all ages) and IDSR (weekly under-5) diarrheal data demographics are shown in Table 2. For the outpatient data, diarrheal outbreak patterns were similar in both under-5 children and in older children and adults, with diarrheal disease in individuals ≥ 5 years representing almost half of the reported cases. Significant differences in diarrhea type were identified when outpatient data were stratified by age (Table 3). However, more than half of the diarrheal cases across age groups presented with some or severe dehydration or bloody diarrhea (Table 3).

**Table 2:** Demographic distributions of outpatient diarrhea cases and IDSR under-5 diarrhea cases in Chobe District.

Attribute	Outpatient	IDSR
**Age (years)**		
<1	29.5	N/A
1–4	27.0	N/A
5+	43.5	N/A
**Male sex**	48.5	N/A
**Diarrhea type**		
No dehydration	34.6	46.5
Some dehydration	55.1	39.0
Severe dehydration	3.7	6.6
Bloody diarrhea	6.7	7.5

IDSR data were not stratified by age or sex. Table entries represent percent of total cases in that category.

IDSR, Integrated Disease Surveillance and Response; N/A, not applicable.

**Table 3:** Outpatient diarrhea cases stratified by age and type of diarrhea.

Diarrhea type	Age (years)			Chi-squared *p*-value
	<1	1–4	5+	
No dehydration	31.6	37.7	34.6	<0.001
Some dehydration	57.8	52.4	55.0	<0.001
Severe dehydration	4.3	3.9	3.1	0.004
Bloody diarrhea	6.3	6.0	7.3	0.016

Entries represent percentage of total cases in that age group. *p*-Values are calculated using a chi-squared test for differences in proportions across groups.

Outpatient diarrheal demographics also differed significantly between the wet and dry seasons (Table 4). While no difference in sex distribution could be detected, the wet season had significantly more patients between the ages of 1 and 4 years (*p <* 0.001), and the dry season had significantly more patients 5 years and over (*p <* 0.001). There was no seasonal difference in the number of diarrhea case reports for children less than 1 year old. Diarrheal type also varied by season (*p <* 0.001).

**Table 4:** Demographics by season of outpatient diarrhea cases in Chobe District.

Attribute	Wet season	Dry season	Chi-squared *p*-value
**Age (years)**			
<1	29.2	29.7	0.524
1–4	29.2	25.6	<0.001
5+	41.6	44.7	<0.001
**Male sex**	49.3	48.1	0.143
**Diarrhea type**			
No dehydration	39.4	31.4	<0.001
Some dehydration	49.5	58.8	<0.001
Severe dehydration	2.7	4.3	<0.001
Bloody diarrhea	8.3	5.6	<0.001

Table entries represent percent of total cases in that season. *p*-Values were calculated using a chi-squared test.

### Hydrometeorological and water quality variables

Average maximum temperature was similar in the wet season and dry season, at 30 °C and 29 °C, respectively; however, maximum temperature had greater variability during the dry season. Minimum temperature was higher during the wet season on average (19 °C) than during the dry season (12 °C). The lowest minimum temperatures occurred during the dry season, and then temperatures increased up to the start of the wet season, at which point they plateaued. In this system, with the commencement of the flood pulse, the height of the Chobe River steadily increased during the wet season, peaking on average in April and decreasing through the dry season.

Chobe River measurements of *E*. *coli* and TSS from 2011 to 2017 also exhibited seasonal patterns (Fig 2F and 2G; full time series data are provided in S1 Fig). TSS increased and peaked during the dry season, declining steadily throughout the wet season. In contrast, *E*. *coli* concentrations were relatively low during the dry season and peaked in the middle of the wet season.

### Environmental predictors of under-5 diarrhea

#### Dry season multimodel results

**Fig 3:**
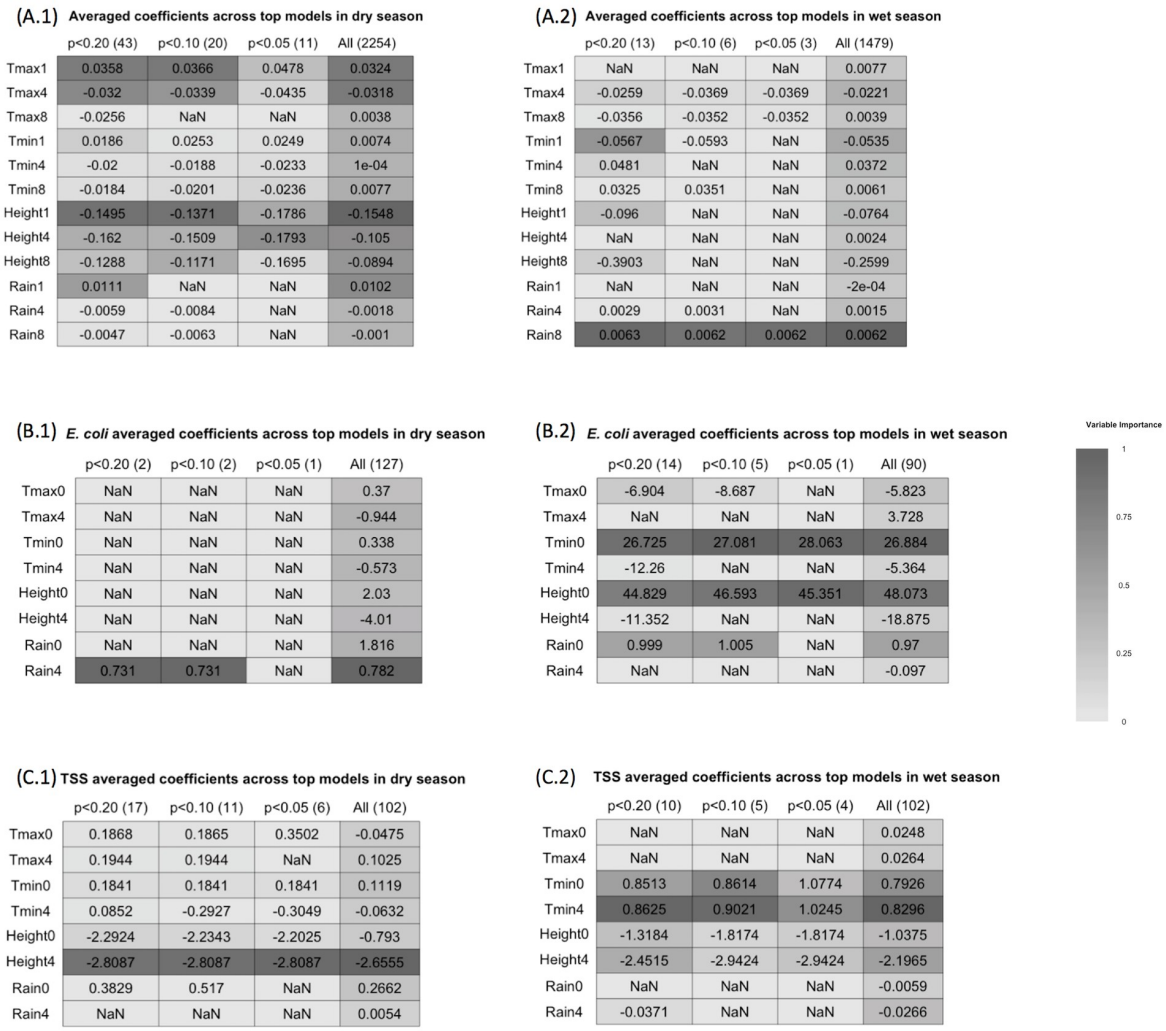
Dry season and wet season average coefficients and variable importance. (A) Summary of multimodel inference predicting under-5 diarrhea. The rows of the tables represent each environmental variable at 1-, 4-, and 8-week lags. The columns of the tables represent different model selection criteria, i.e., average coefficient estimates for each environmental variable derived from different model subsets. The numbers in parentheses indicate the number of models that were averaged in a given model subset. Lastly, the shading indicates the weighted importance of each variable within the model subset, with 1 being the highest possible weighted importance. “NaN” indicates that a variable was not used in any of the models within a model subset. (A.1) shows results from the dry season, and (A.2) shows wet season results. (B and C) As for (A), but for models predicting *E*. *coli* and total suspended solids (TSS), respectively, with 0- and 4-week lags. Tmax, maximum temperature; Tmin, minimum temperature.

The results from multimodel inference in the dry season are summarized in Fig 3A.1. River height lagged 1 week, and maximum temperature lagged 1 and 4 weeks had the highest weighted importance within all model subsets. Indeed, average coefficient estimates and variable importance did not differ greatly between model subsets with different *p*-value criteria. River height lagged 4 weeks and rainfall lagged 1 week also had moderate relative importance when rainfall—rare in the dry season—occurred.

As the Chobe River height decreases during the dry season, floodwaters drain back into the channel from the surrounding floodplains. Multimodel inference estimates indicated that a 1-meter decrease in river height in the dry season was associated with a 16.7% (1-week lag) and a 16.1% (4-week lag) increase in under-5 diarrhea cases. Maximum temperature had a more complex relationship with diarrheal disease, with a positive influence on diarrheal disease initially at the 1-week lag but a negative relationship when lagged by 4 weeks.

A 1 °C increase in maximum temperature lagged 1 week was associated with an estimated 3.8% increase in under-5 diarrhea, while a 1 °C decrease in maximum temperature lagged 4 weeks was associated with a 3.4% increase in under-5 diarrhea.

Pseudo *R*-squared values for the averaged models ranged from 0.55 to 0.57 (S1 Table), while the null model (with only an autoregressive term and year dummy variable) had a pseudo *R*-squared value of 0.46. Prediction results for different model averages were consistent, so this discussion focuses on the model subset with coefficients significant at *p <* 0.05. LOO cross-validation prediction accuracy was typically higher for the weighted averaged models than the top AICc model but varied greatly depending on the omitted year (S2 Table). Correlations between predicted number of cases of under-5 diarrhea and observed number of cases were very high for 2009 through 2017, but the 2008 correlation was very poor (*r* = 0.1214). The averaged models containing environmental predictors performed much better overall in LOO cross-validation than the null model (S4 Table).

#### Wet season multimodel results

Average coefficients and variable importance estimates in the wet season also did not vary widely between model subsets (Fig 3A.2). Rainfall lagged 8 weeks was the most important predictor of under-5 diarrhea in the wet season. Each additional 10 mm of rainfall lagged 8 weeks was associated with a 6.5% increase in under-5 diarrhea incidence. Minimum temperature lagged 1 week had moderate importance in 2 model averages, which estimated a 5.8% increase in diarrhea for every 1 °C decrease.

Pseudo *R*-squared values for the wet season were 0.54 for all model averages, and the null model had a value of 0.49 (S1 Table). All model averages produced similar LOO prediction accuracy; models with coefficients significant at *p <* 0.05 are provided S2 Table. Similar to the dry season models, prediction accuracy varied greatly based on the omitted year of data. Correlations from model averaged predictions were higher than 0.80 for the years 2010–2014 and 2016. When data from 2015 were omitted, the models generated very poor predictions. However, the null model performed worse on average in LOO cross-validation than the averaged models with environmental predictors.

We also tested the data using distributed lag nonlinear models, which allow for nonlinear relationships between predictors and the outcome variable. All environmental variables were included as predictors (maximum temperature, minimum temperature, rainfall, and river height) lagged from 0 to 8 weeks. The results generally agreed with the results presented above. In the dry season, decreasing river height was associated with a significantly higher risk of diarrheal disease at lag weeks 4–6. In the wet season, higher rainfall was associated with larger diarrhea risk at lag weeks 7 and 8. Associations between other environmental variables and diarrhea incidence were generally nonsignificant, which may be due to our moderate sample size. Of note, the estimated exposure—response relationships were relatively linear, which supports our use of generalized linear model multimodel inference above. More details and figures explaining this analysis can be found in S1 Text and S6 and S7 Figs.

### Environmental predictors of TSS and *E*. *coli*

Multimodel inference identified different environmental drivers of *E*. *coli* and TSS by season (Fig 3B and 3C). In the wet season, minimum temperature unlagged, river height unlagged, and rainfall unlagged were the most important predictors of *E*. *coli* concentrations. All 3 variables had statistically significant positive associations with *E*. *coli* (Fig 3B.2). Rainfall lagged 4 weeks was the only important predictor of *E*. *coli* in the dry season and had a positive coefficient (Fig 3B.1). Rainfall in the dry season, however, was an uncommon event.

In the dry season, TSS level was strongly predicted by river height lagged 4 weeks, which had a negative coefficient (Fig 3C.1). Lastly, minimum temperature lagged 0 and 4 weeks and river height lagged 4 weeks were important predictors of TSS in the wet season. Minimum temperature at both lags had a positive association with TSS, and river height had a negative association (Fig 3C.2).

### Water quality and diarrheal case reports

Univariate regression analyses showed that both TSS and *E*. *coli* concentrations were significantly associated with under-5 diarrheal disease (S2 Fig). TSS had a positive association with diarrhea incidence in the dry season unlagged (beta = 0.060, 95% CI = 0.180, 0.102), lagged 3 weeks (beta = 0.041, 95% CI = 0.008, 0.073), and lagged 4 weeks (beta = 0.042, 95% CI = 0.004, 0.080). In contrast, TSS had a significantly negative relationship with under-5 diarrhea in the wet season lagged 2 weeks (beta = −0.098, 95% CI = −0.151, −0.045) and 6 weeks (beta = −0.063, 95% CI = −0.119, −0.007). *E*. *coli* had an unlagged positive association with diarrheal incidence in both the wet season (beta = 0.003, 95% CI = 0.001, 0.004) and the dry season, associated with rare rainfall events (beta = 0.011, 95% CI = 0.003, 0.017).

## Discussion

### Outbreak patterns and seasonal influences on demographic and clinical characteristics of diarrhea case reports

In this system, the presence of surface water and flood pulse dynamics in a river—floodplain system had a significant association with diarrheal disease case reports in children under 5 years of age as well as all other age groups reporting to health facilities. Timing of seasonal outbreaks differed from that observed at the level of the country, where surface water is largely absent [25], highlighting the important influence landscape characteristics can have on disease dynamics and population vulnerability. In this study, diarrheal incidence was highest on average in the dry season in August, with the second outbreak occurring in the wet season towards the end of January. Importantly, the timing of outbreaks was similar across age groups. Important seasonal differences were identified in the average number of diarrheal cases reported for the age groups 1 to 4 years and 5 years and over (*p <* 0.001; Table 4). This was not the case, however, for children less than 1 year of age. The reasons for this remain unclear but may relate to lower immunocompetence in the youngest children relative to the other age groups and greater general susceptibility to waterborne pathogen communities across seasons. Diarrhea type also varied significantly by season (*p <* 0.001; Table 4), with, for example, bloody diarrhea occurring more frequently in the wet season, suggesting that environmental conditions influence pathogen community and exposure dynamics, associations that have been previously reported [28].

It is important to note, however, that passive surveillance data have potential limitations influenced by health seeking behaviors and other critical sociocultural drivers, and data may, therefore, not fully represent the population under study (for a full review see [25]). Additional community-level studies are important and can provide insight into potential infection dynamics not captured by passive surveillance systems [29]. Nevertheless, the season—diarrhea interactions observed here underscore the important role environmental drivers may have for predicting the timing of disease outbreaks as well as the demographic and clinical characteristics of patients affected by season.

### Environmental drivers of diarrheal disease

**Fig 4:**
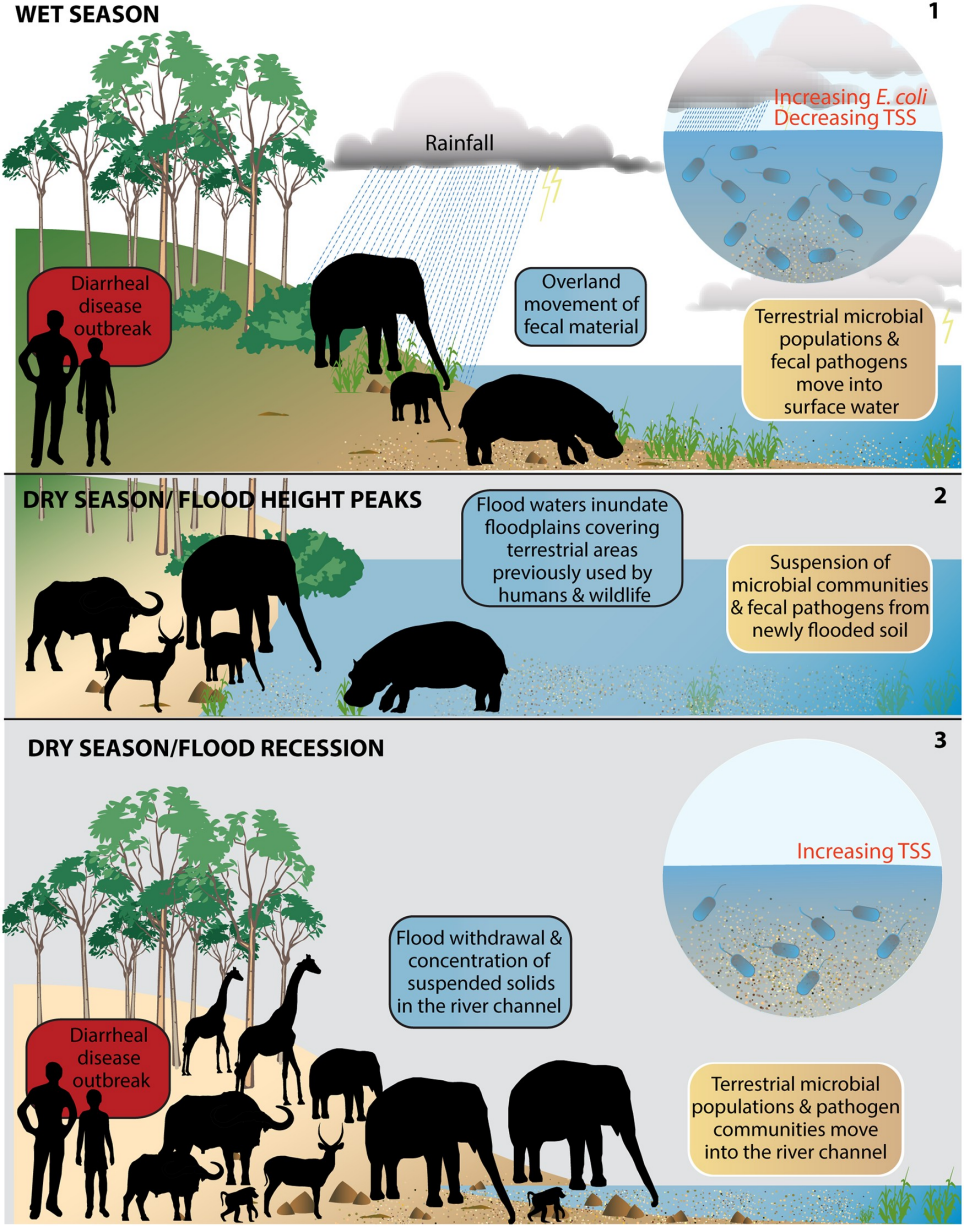
Schematic of rainfall and flood pulse influences on surface water quality and diarrheal disease outbreaks in a flood pulse river system. In these systems, the flood pulse uniquely links aquatic and terrestrial habitats and microbial communities including potential pathogens. During the wet season (panel 1), rainfall moves fecal *E*. *coli* (a marker for fecal bacteria) overland from riparian areas into the river channel. As in-river *E*. *coli* increases, the number of diarrheal disease case reports increases in the population using municipal water that was obtained from the river (wet season outbreak). During this same period, seasonal pans are filled with rainfall, and water-dependent animals (wildlife and, to a lesser extent, livestock) move out of the riparian area into the interior to utilize forage and water resources. Floodwaters (panel 2) rise and inundate floodplains, incorporating fecal microbial communities on previously dry land areas. With progression of the dry season and no rainfall, water pans in the interior dry up, and water-dependent animals move back to the river’s edge, concentrating fecal material along river floodplains while utilizing the only permanent surface water in the system. Floodwaters begin to recede from the inundated land areas (panel 3), and TSS levels increase, as does the number of diarrheal case reports (dry season outbreak). In the rare event of rainfall in the dry season, *E*. *coli* levels in the water channel increase and are positively associated with diarrheal case reports (not shown). TSS, total suspended solids.

In this flood pulse system, diarrheal disease was strongly coupled to environmental drivers (Fig 2). Across tropical and semiarid regions [25,30,31], rainfall has been identified as a critical predictor of diarrheal disease. Consistent with these studies, rainfall with an 8-week lag had a significant influence on under-5 diarrhea, with a 10-mm increase in rainfall associated with an estimated 6.5% rise in the number of cases reported. Rainfall, minimum temperature, and river height were all associated with increases in *E*. *coli* in the river system (Figs 3, 4 and S2). TSS, however, had a negative association with diarrheal cases in the wet season, with in-river levels declining as river height rose in conjunction with the arrival of floodwaters (lagged 2 and 6 weeks; S2 Fig). However, significant variation in TSS levels between transect points and river segments by season have previously been noted [26].

The number of diarrheal case reports was higher on average in the dry season (Fig 2) and was linked to maximum temperature and flood height, with a 1-m drop in floodwaters during floodwater recession from floodplains associated with a significant increase in diarrheal cases of 16.7% and 16.1% lagged 1 and 4 weeks, respectively (Figs 3 and S2). River TSS levels were strongly predicted by river height and had a significant positive association with diarrhea in the dry season (lagged 0, 3, and 4 weeks; S2 Fig). Here, as floodwaters receded from surrounding floodplains, TSS levels increased within the river channel. Differences in lagged relationships suggest that hierarchical environmental processes are occurring, where primary source exposure and secondary transmission dynamics may further contribute to variation in the observed timing of environmental—health couplings.

In our analysis, cross-validation results reveal important heterogeneity in the ability of our environmental models to accurately predict diarrheal case reports across the 8-study villages. Diarrheal disease as a syndrome is complex, driven by a variety of pathogens, each varying in their transmission and persistence dynamics in the system, elements that will vary, together with environmental drivers, within and between years. Sociocultural influences will also shape exposures, secondary transmission, and case detection. These complexities are evident in the demographic and clinical variation seen by season in the outpatient data. However, across models, river height lagged 1 week in the dry season and rainfall lagged 8 weeks in the wet season remained consistently important across all years in predicting diarrheal disease case reports.

### Flood pulse systems and microbial dynamics at the aquatic—terrestrial interface

Waterborne pathogen dynamics are closely coupled with hydrological and ecological processes across the aquatic—terrestrial interface and consequent human microbial exposure risk [19,32]. In the studied system, the flood pulse played a critical role in outbreak dynamics across age groups. In such flood pulse systems, floodplain environments are periodically flooded with sediment, with fecal microbial transport occurring between the floodplain and the river channel [33], influencing microbial community structure and movement across the aquatic—terrestrial interface (Fig 4) [19,32]. The character of the flood pulse (alternating cycles of increased water flow, volume, inundation, and draining) establishes the degree of connectivity, with lateral exchange of matter and microbial organisms including waterborne pathogens moving across hydrological connectivity gradients [20,21,34,35]. Pathogen communities are shaped by the interactions that occur at this aquatic—terrestrial interface and influence the character of waterborne pathogen exposures in associated populations.

Typically, human health impacts are associated with the rise in water levels and advancement of floodwaters, although the timing of this relationship can be complicated [15,36]. However, in the Chobe River system, flood recession and withdrawal from the floodplains back into the river channel drives diarrheal disease outbreaks (Fig 4). Cholera in the Bengal Delta region provides an important contrasting example. In this system, cholera outbreaks occur biannually, a pattern that is unique to this region [37]. Here, the flood pulse is important in distributing the cholera pathogen, with disease outbreaks corresponding to high flood levels and water movement distributing pathogens across floodplains. This contrast suggests that differences in pathogen reservoirs (aquatic versus terrestrial) play a critical role in the timing of diarrhea—flood relationships and human exposure dynamics. Advancing our understanding of these human—environment couplings will provide critical insight into forecasting flood—waterborne disease dynamics across different landscapes—including for emerging diseases—by pathogen type.

### Public health implications

#### Under-5 diarrhea surveillance focus

While significant differences were identified in diarrhea type by age, moderate and severe diarrheal disease (diarrhea with some or severe dehydration or bloody diarrhea) constituted greater than half of the reported cases for all age groups (>60%; <1, 1–4, 5+ years; Table 3). The study region has one of the highest HIV infection rates in the country (male, 14.4%; female, 18.1% [38]), raising concerns regarding the potential immunosuppressive role of this disease in creating increased vulnerability in individuals 5 years of age and over. A public health focus on diarrheal disease control in children under 5 years of age may, therefore, be inadequate in populations with high HIV burdens, where additional public health interventions may be required.

#### Flood pulse systems and impacts of suspended solids on the production of safe drinking water

In developing countries, much attention has been focused on household treatment of water as a primary public health intervention for diarrheal disease [39]. While this is critically important, it is an intermediate solution. Many of these regions have water treatment plants that are operational, but clean water is not consistently available. In this study, households in the 8 villages had access to and reported utilizing improved water produced by centralized water processing plants (97% [40]). This infrastructure should protect the population from in-river water quality declines and waterborne disease, yet biannual diarrheal outbreaks continue to occur, coinciding with degraded river water quality. While numerous factors may influence the ability of water treatment and distribution systems to provide clean water to a population, hydrological variability as observed in this system across seasons can create extreme shifts in TSS levels (which include natural organic material [NOM]), alkalinity, and pH, presenting a challenge to conventional water treatment processes.

Suspended solids have an important influence on bacterial survival and transport in surface waters, providing protection from UV light, predators, and grazers, and access to nutrients (reviewed in [41,42]). Similarly, enteroviruses within surface water bodies are more commonly attached to suspended solids [43]. Transport and deposition of microbial contamination can also be influenced by the type of sediment particles present in surface waters, with significantly greater adsorption of *E*. *coli* reported for soils with higher clay content [44]. Clay soil has a high number of pore spaces, which provide niches for microbes and retain moisture and nutrients, while the small average size of these pores reduces *E*. *coli* mortality by conferring protection from predation by larger-bodied nematode soil fauna [45,46].

Elevations in suspended solids not only influence microbial survival in the river but also through the water treatment facility if these elevations are not detected and accounted for in the treatment process. Conventional water treatment includes a coagulation step, which involves the application of chemicals to remove suspended solids (sediments and NOM) from the water into large floc aggregates that are subsequently eliminated through sedimentation and filtration steps [47]. The effectiveness of these steps is influenced by water pH, alkalinity, and NOM levels in the water [29]. This is then followed by a chemical disinfection step, most frequently through chlorination.

Many treatment facilities in Africa and elsewhere, however, rely on manual control strategies to determine the coagulation doses needed for water treatment [48], amounts that may be grossly inadequate during periods of rapid hydrological change and shifts in upstream TSS levels and other water properties [49]. Even when elevations in TSS levels are detected, calculation of the optimum coagulant dose is complex and nonlinear, further complicating successful application of manual approaches [50,51]. TSS elevations can occur rapidly, varying over months, weeks (S1 Fig), days, and even within a day (in this system >43% increase observed over 8 hours just above the water treatment plant inflow [one-time observation]; S5 Table), with pH and alkalinity changes also influencing chemical effectiveness. Failure of the operator and/or equipment in water treatment plants to detect and account for these fluxes when applying chemical dosing can compromise water quality as the coagulation step has crucial influence on disinfection and microorganism removal [47,51,52]. Microbial growth can also continue beyond the water treatment facility in the water distribution system [53], particularly when water shortages and low water pressure increase water stasis in the system. River system degradation will influence these dynamics even further through additional inputs from erosion and runoff or surface water disturbances such as boat traffic [49,54].

#### Flood pulse systems, diarrheal disease, and climate change

Under climate change, rainfall variability is expected to increase across Southern Africa in the 21st century, with a spread in the rainfall probability distribution, increasing the frequency of droughts, extreme rainfall events, and floods in the future [55]. In flood pulse systems, existing hydrological dynamics already create significant variation in the volume of water flow in river systems and the area of floodplain inundation (401 km^2^ to 5,779 km^2^ from 2000 to 2015 in the Chobe River system [56]), with consequent impacts on water quality dynamics and waterborne pathogen exposure. Predicted increases in hydrological extremes associated with climate change will further elevate the vulnerability of populations dependent on surface water resources in these landscapes.

#### Predicting climate—health interactions in flood pulse systems

Flood pulse dynamics in the Chobe River arise from precipitation events in catchment headwaters more than 1,000 miles away and determine the magnitude, duration, and timing of river flow and consequent effects on dry season diarrheal disease in downstream populations. Climate controls on hydrometeorological conditions can vary significantly across a watershed. For example, rainfall in the Angolan Highlands catchment area is driven by the Intertropical Convergence Zone and Congo Air Boundary even as more local circulation changes contribute to the increasingly arid conditions experienced in Botswana in the interior of Southern Africa. Distant meteorological events and teleconnections associated with, for instance, the Walker circulation can further influence diverging climate dynamics (reviewed in [57]). As these hydrometeorological processes are intimately linked with diarrheal disease incidence, we can expect shifts in diarrheal disease patterns to be responsive to both local and distant climate dynamics and associated changes under climate change. It is evident that exclusive use of local or country-level climate data would be inadequate to forecast climate impacts on diarrheal disease in this region. It is further evident that clumping regions into larger diarrheal disease studies likely masks critical information necessary to understand environmental—health relationships and climate vulnerabilities.

#### One health approach to understanding environmental drivers of diarrheal disease

**Fig 5:**
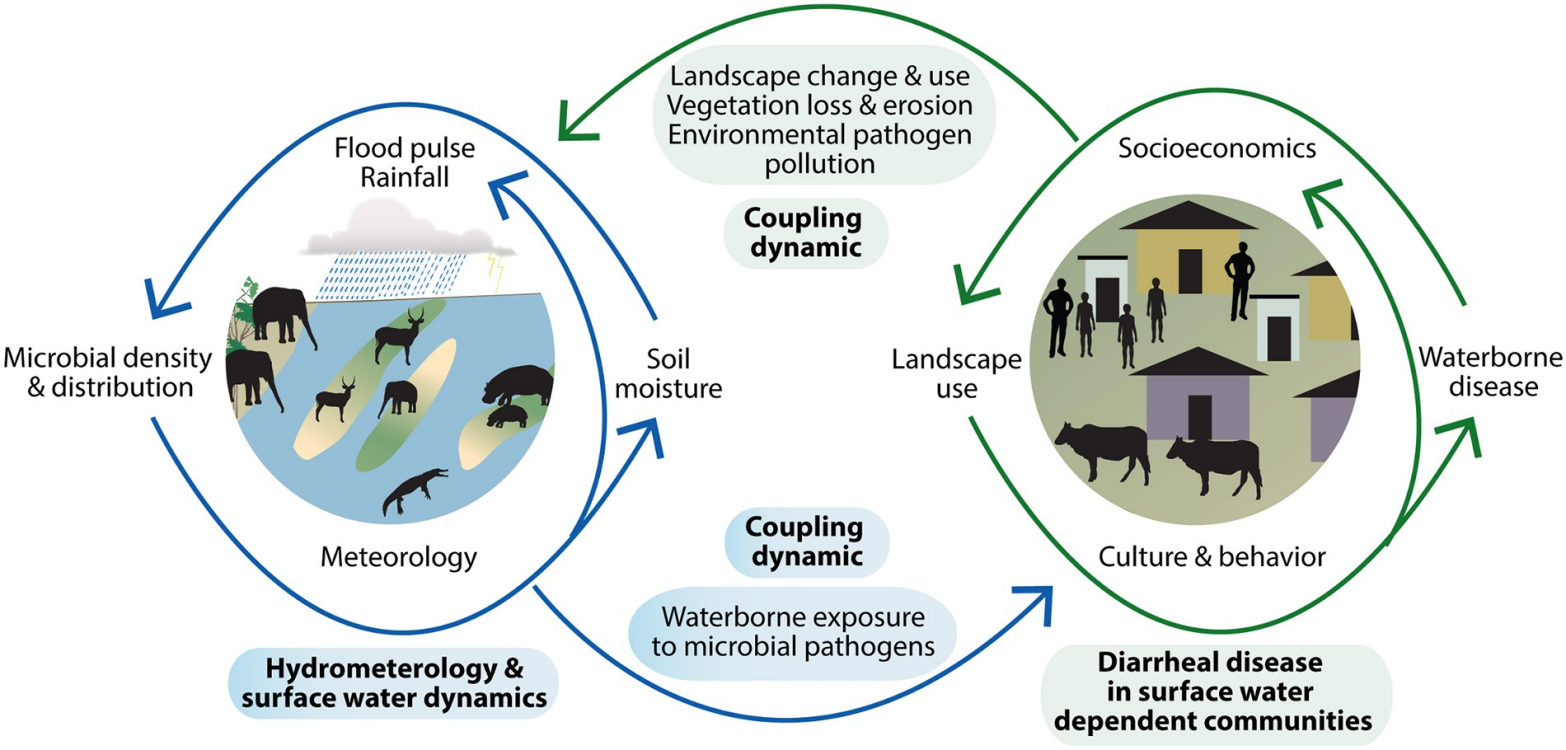
Schematic of linked processes across the aquatic—terrestrial interface in a flood pulse river—floodplain system like the Chobe River in Botswana. Hydrological, geomorphological, sedimentary, and ecological processes influence fecal microbial dynamics, water quality, and human waterborne disease risk in surface-water-dependent populations. These interactions are coupled to sociocultural and economic processes, influencing landscape change and exposure to waterborne disease, feeding back to further environmental degradation and pathogen pollution potential.

Pathogen-specific transmission pathways and host exposure and infection dynamics interact in particular ways with environmental and socioecological variables to influence the occurrence of diarrheal disease across populations and landscapes, predominantly in regions of poverty, where environmental exposures are enhanced, as is vulnerability to climate change (Fig 5). These interdependent influences complicate simple climate—diarrheal disease evaluations, affecting the development of sustainable interventions. While critical hydrometeorological drivers were associated with in-river water quality declines and diarrheal disease outbreaks in this study, this is only part of the story. For example, our previous work in this system has identified significant associations between the spatial and temporal patterns of dry season *E*. *coli* and TSS increases, protected land use, floodplain habitat, and riparian fecal counts from elephant and other wildlife [26]. These elements were also predictive of the spatial occurrence of in-river *E*. *coli* concentrations in the early wet season above the water treatment plant intake.

These findings identify important couplings between flood pulse dynamics, seasonal fluxes in wildlife densities, land use, and water quality, with impacts on safe drinking water and diarrheal disease in children and adults. The complexity of the couplings in this system underscore the need for the inclusion of dimensions not normally considered part of the domain of public health. This is particularly true for emerging infectious diseases influenced by environmental processes and consequently expected to be impacted by climate change.

### Conclusions

From dryland to tropical systems, surface water plays a critical role in human health. Floods and rainfall variability are expected to increase under climate change across many systems and can induce dramatic shifts in surface water quality. While presenting a clear risk in populations using unsafe water sources, rapid change in suspended solids and other water quality parameters may overwhelm conventional water treatment processes, impacting the provision of safe drinking water and increasing the potential for waterborne pathogen exposure and diarrheal disease. Long-term solutions to diarrheal disease and climate preparedness should include increased national focus on water sector development using technologies that are robust to local environmental conditions but can be serviced and maintained locally. In populations with high HIV burdens, expansion of diarrheal disease surveillance and intervention strategies might be needed, engaging other at-risk sectors beyond the under-5 age group. Ultimately, the success of public health strategies across systems will depend on understanding how sociocultural and environmental factors are coupled across scales and influence waterborne disease exposure risk, transmission dynamics, and population vulnerability in the face of expected climate change.

## Supporting information

S1 Fig Time series of diarrhea, environmental, and water quality data from 2007 to 2017. (TIF)

S2 Fig Coefficient estimates for the effect of TSS and *E*. *coli* on under-5 diarrhea. Each point represents the regression coefficient for the respective water quality measure at a different lag week. The dotted lines provide the 95% confidence intervals for the corresponding coefficient estimate.
(TIF)

S3 Fig Dry season average coefficients and variable importance using standardized diarrhea data. Summary of multimodel inference predicting under-5 diarrhea (standardized). The rows of the table represent each environmental variable at 1-, 4-, and 8-week lags. The columns of the table represent different model selection criteria, i.e., average coefficient estimates for each environmental variable derived from different model subsets. The numbers in parentheses indicate the number of models that were averaged in a given model subset. Lastly, the colors indicate the weighted importance of each variable within the model subset, with 1 being the highest possible weighted importance. “NaN” indicates that a variable was not used in any of the models within a model subset.
(PNG)

S4 Fig Wet season average coefficients and variable importance using standardized diarrhea data. Summary of multimodel inference predicting under-5 diarrhea (standardized). The rows of the table represent each environmental variable at 1-, 4-, and 8-week lags. The columns of the table represent different model selection criteria, i.e., average coefficient estimates for each environmental variable derived from different model subsets. The numbers in parentheses indicate the number of models that were averaged in a given model subset. Lastly, the colors indicate the weighted importance of each variable within the model subset, with 1 being the highest possible weighted importance. “NaN” indicates that a variable was not used in any of the models within a model subset.
(TIF)

S5 Fig Coefficient estimates for the effect of TSS and *E*. *coli* on under-5 diarrhea (standardized). Each point represents the regression coefficient for the respective water quality measure at a different lag week. The dotted lines provide the 95% confidence interval for the corresponding coefficient estimate.
(TIF)

S6 Fig Nonlinear exposure—response relationships between river height and diarrhea incidence in the dry season. These relationships were generated using distributed lag nonlinear models that controlled for minimum temperature, maximum temperature, rainfall, and year. Relative risk estimates (black lines) and confidence intervals (grey areas) are shown across different river height levels (*x*-axis). All relative risks are in reference to a river height increase of 6 m. (A—H) represent the response functions at lag weeks 1 through 8. We can see from (D—F) that declines in river height at lag weeks 4–6 are associated with increased risk of diarrheal disease.
(TIF)

S7 Fig Nonlinear exposure—response relationships between rainfall and diarrhea incidence in the wet season. These relationships were generated using distributed lag nonlinear models that controlled for minimum temperature, maximum temperature, rainfall, and year. Relative risk estimates (black lines) and confidence intervals (grey areas) are shown across different rainfall levels (*x*-axis). All relative risks are in reference to rainfall of 0 mm. (A—H) represent the response functions at lag weeks 1 through 8. We can see from (G) and (H) that increases in rainfall at lag weeks 7 and 8 are associated with increased risk of diarrheal disease.
(PNG)

S1 Table Pseudo *R*-squared values for each averaged model in the dry and wet seasons. (XLSX)

S2 Table LOO cross-validation results for the dry season and wet season using correlation as the metric of prediction accuracy. The rows represent the seasonal data and the model structure (i.e., averaged model with environmental predictors or null model without environmental predictors) used to fit the model. The columns indicate the omitted year, which was also used to test model prediction accuracy. Table values represent the correlation of the averaged model prediction with the observation for the omitted year.
(XLSX)

S3 Table Pseudo *R*-squared values for each averaged model in the dry and wet seasons, using standardized diarrhea case data. (XLSX)

S4 Table LOO cross-validation results for the dry season and wet season using the standardized under-5 diarrhea data and correlation as the metric of prediction accuracy. The rows represent the seasonal data that were used to fit the model. The columns indicate the omitted year, which was also used to test model prediction accuracy. Table values represent the correlation of the averaged model prediction with the observation for the omitted year.
(XLSX)

S5 Table Measurement of TSS over an 8-hour period in the Chobe River on transect 33, which is above the intake for the water treatment plant. (XLSX)

S1 Text Comparative analysis with distributed lag nonlinear models. (DOCX)

S1 STROBE Checklist (DOCX)

## Abbreviations

IDSR: Integrated Disease Surveillance and Response
LOO: leave-one-out
MoH: Ministry of Health
NOM: natural organic material
TSS: total suspended solids
