# Supplementary figures and images for: Hydrometeorology and flood pulse dynamics drive diarrheal disease outbreaks and increase vulnerability to climate change in surface-water-dependent populations: A retrospective analysis
Source: PLoS Med. 2018 Nov 8;15(11):e1002688. doi: 10.1371/journal.pmed.1002688 (PMC6224043; doi:10.1371/journal.pmed.1002688)

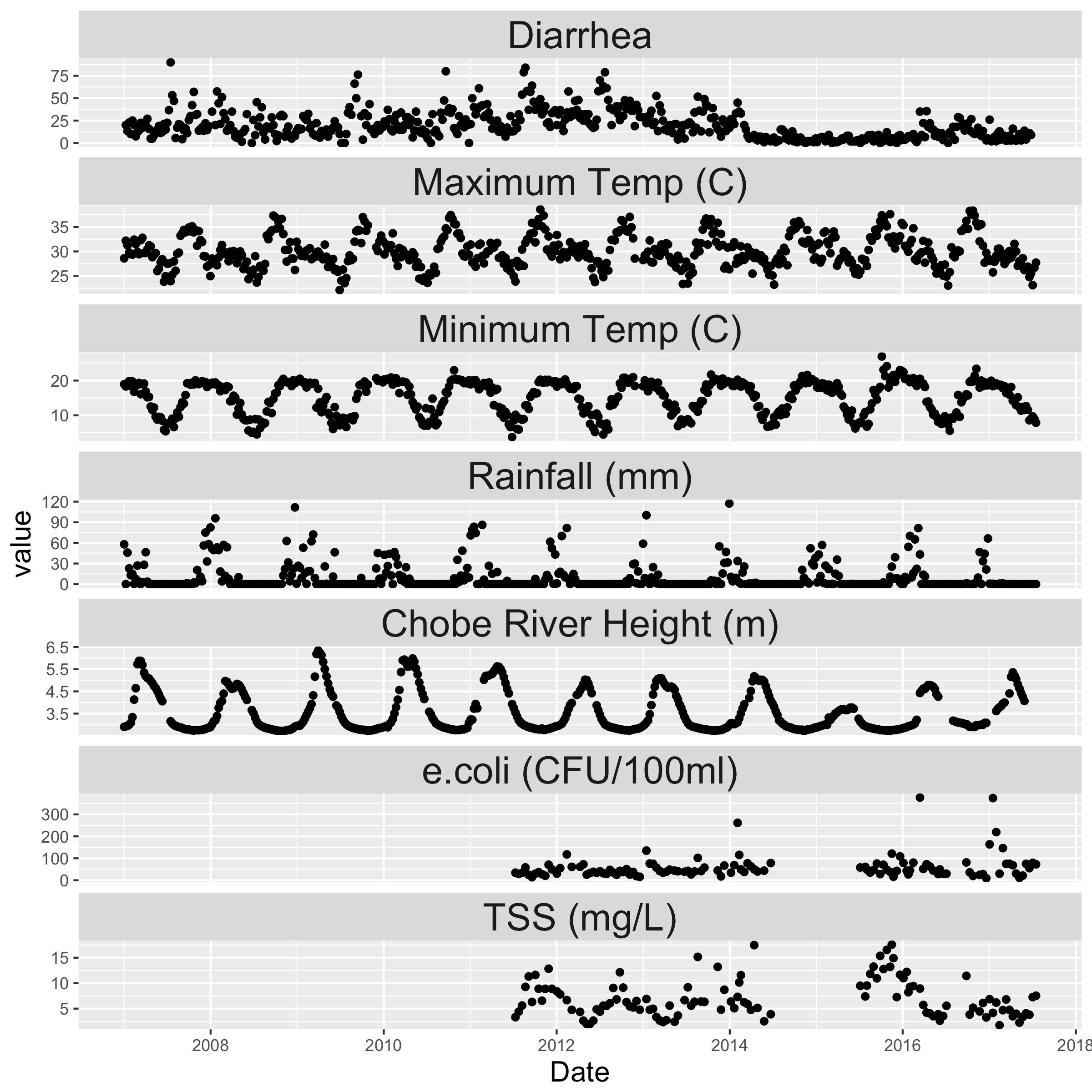

Supplement: S1 Fig — (TIF) [file pmed.1002688.s001.tif]

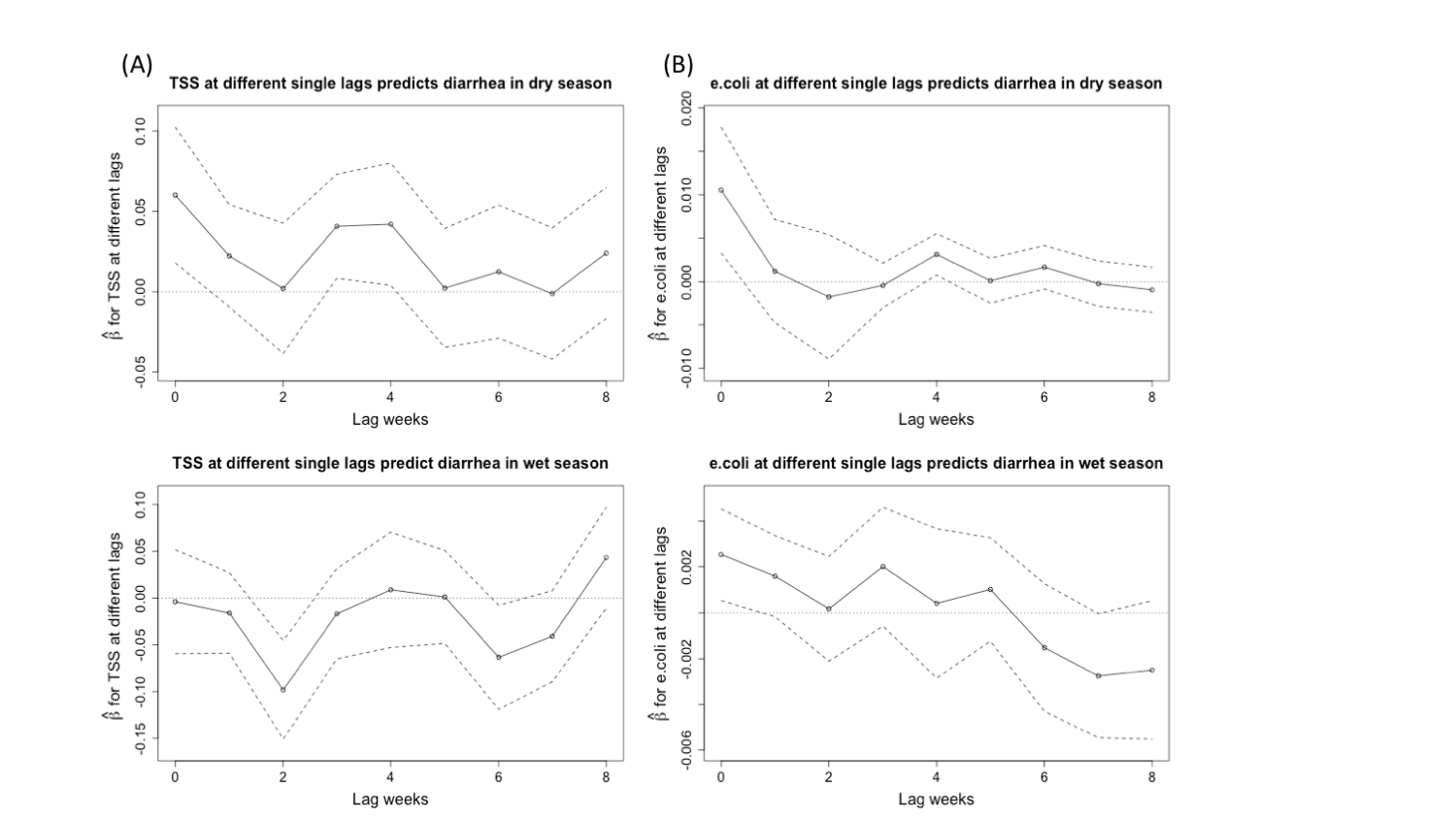

Supplement: S2 Fig — Each point represents the regression coefficient for the respective water quality measure at a different lag week. The dotted lines provide the 95% confidence intervals for the corresponding coefficient estimate. (TIF) [file pmed.1002688.s002.tif]

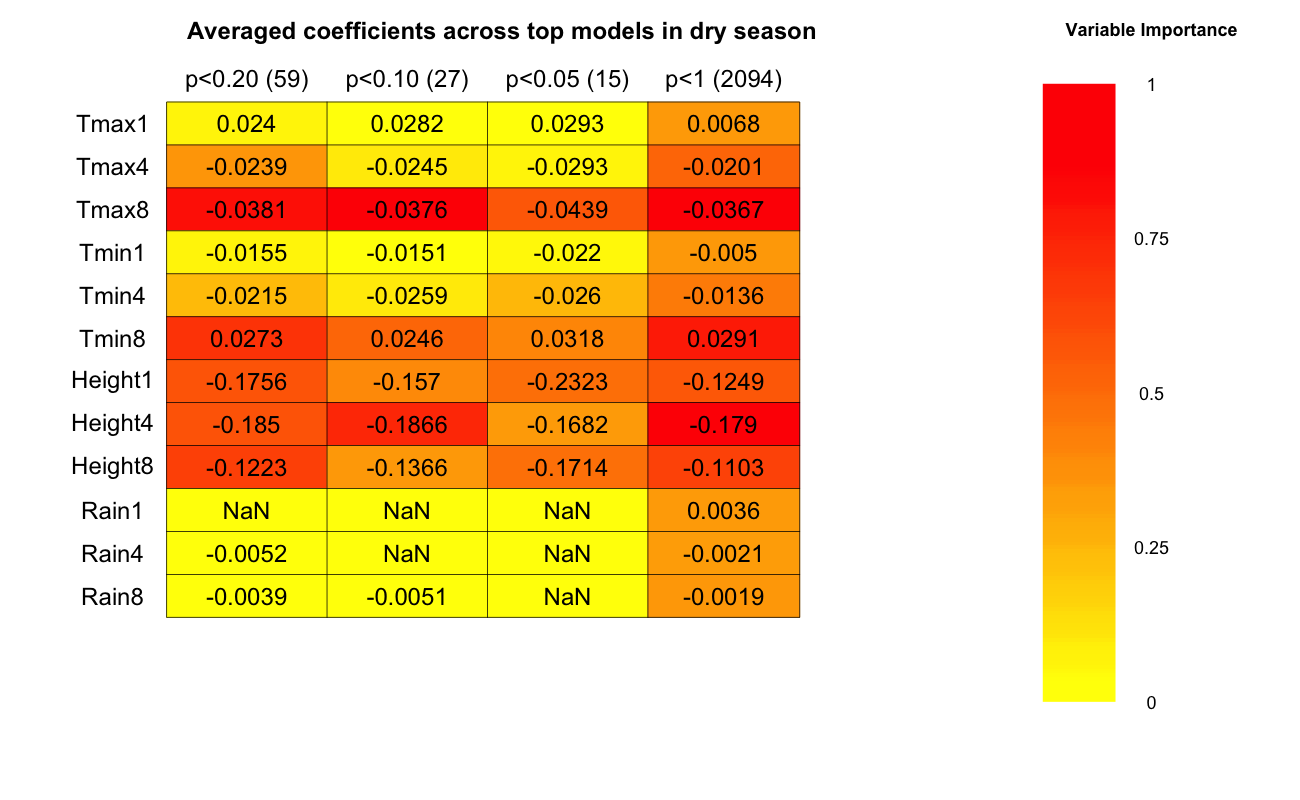

Supplement: S3 Fig — Summary of multimodel inference predicting under-5 diarrhea (standardized). The rows of the table represent each environmental variable at 1-, 4-, and 8-week lags. The columns of the table represent different model selection criteria, i.e., average coefficient estimates for each environmental variable derived from different model subsets. The numbers in parentheses indicate the number of models that were averaged in a given model subset. Lastly, the colors indicate the weighted importance of each variable within the model subset, with 1 being the highest possible weighted importance. “NaN” indicates that a variable was not used in any of the models within a model subset. (PNG) [file pmed.1002688.s003.png]

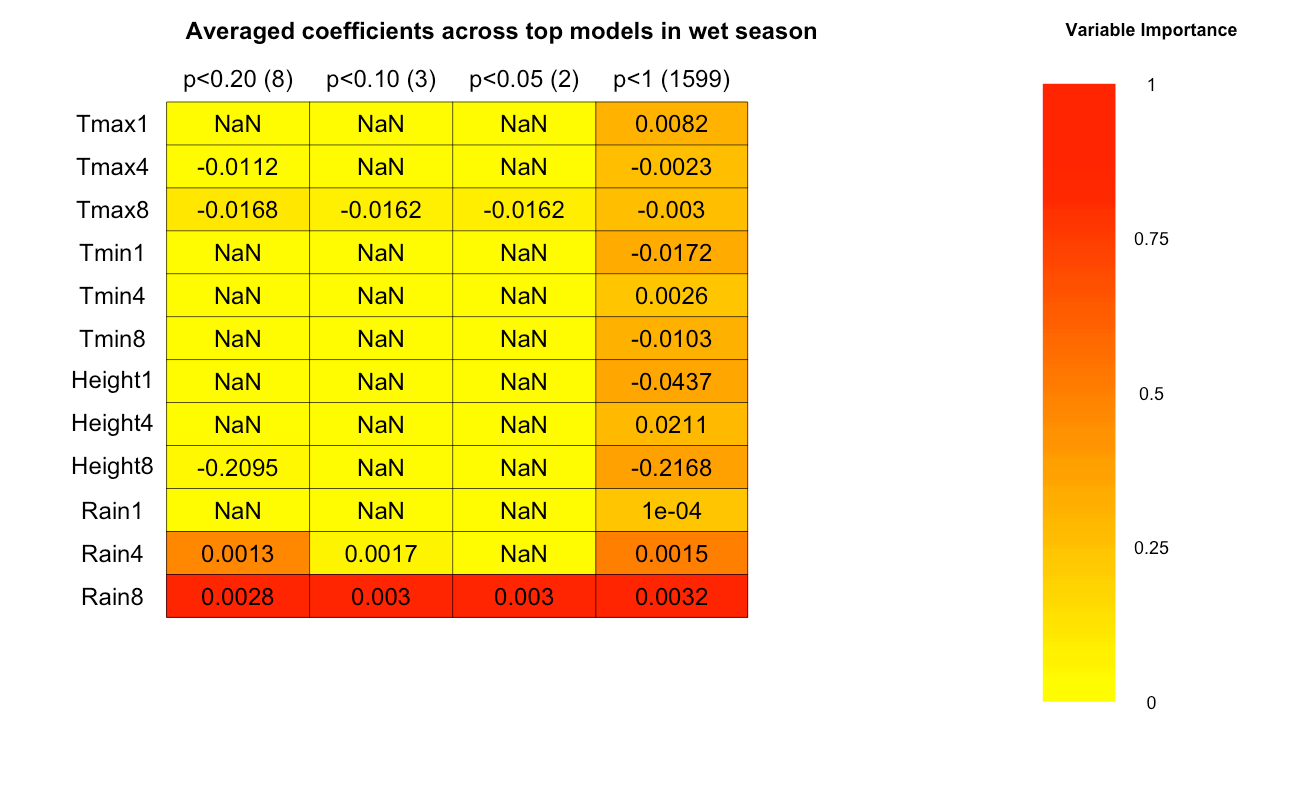

Supplement: S4 Fig — Summary of multimodel inference predicting under-5 diarrhea (standardized). The rows of the table represent each environmental variable at 1-, 4-, and 8-week lags. The columns of the table represent different model selection criteria, i.e., average coefficient estimates for each environmental variable derived from different model subsets. The numbers in parentheses indicate the number of models that were averaged in a given model subset. Lastly, the colors indicate the weighted importance of each variable within the model subset, with 1 being the highest possible weighted importance. “NaN” indicates that a variable was not used in any of the models within a model subset. (TIF) [file pmed.1002688.s004.tif]

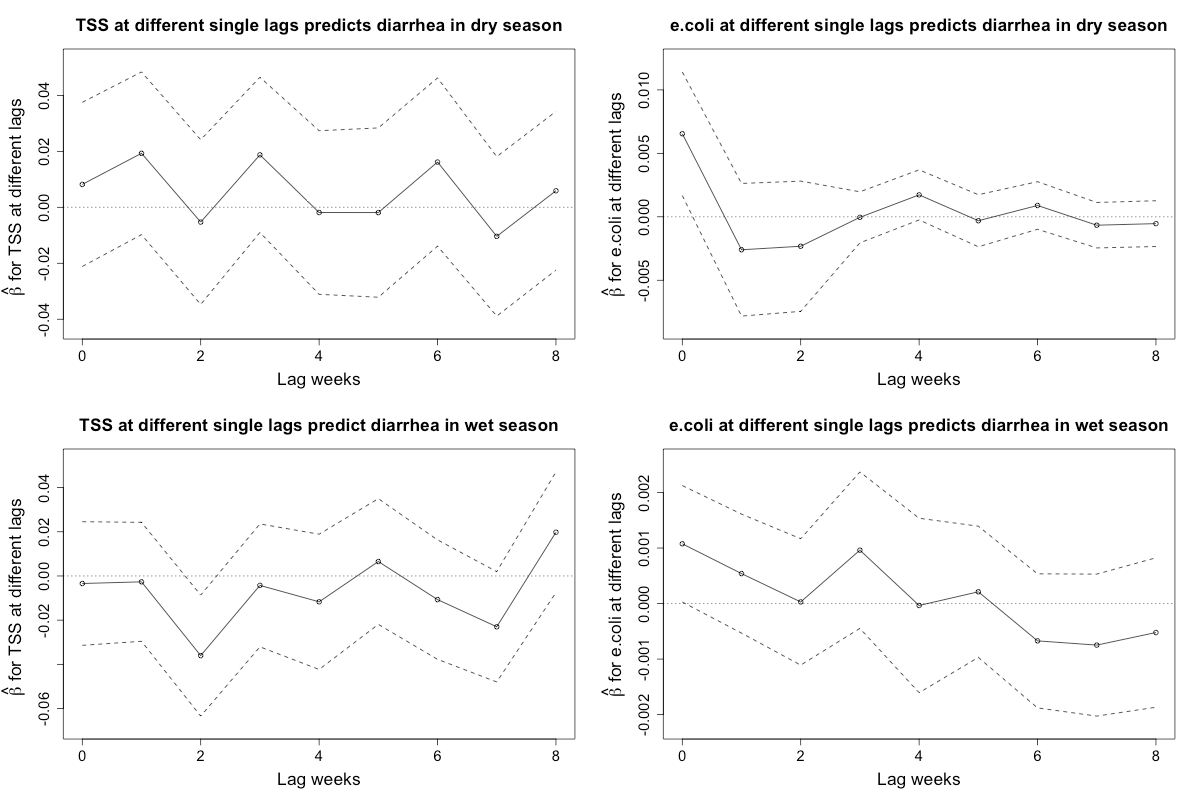

Supplement: S5 Fig — Each point represents the regression coefficient for the respective water quality measure at a different lag week. The dotted lines provide the 95% confidence interval for the corresponding coefficient estimate. (TIF) [file pmed.1002688.s005.tif]

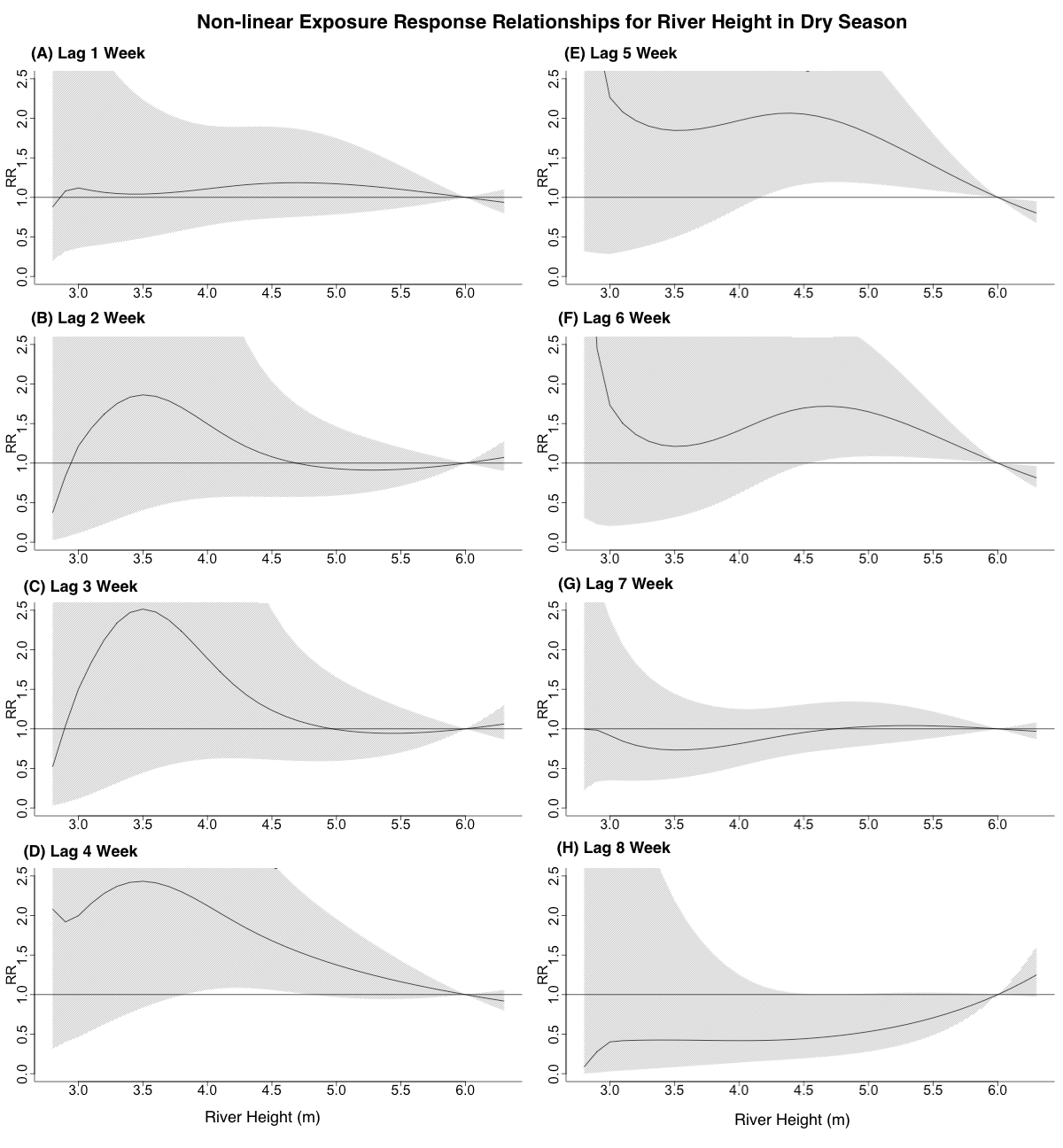

Supplement: S6 Fig — These relationships were generated using distributed lag nonlinear models that controlled for minimum temperature, maximum temperature, rainfall, and year. Relative risk estimates (black lines) and confidence intervals (grey areas) are shown across different river height levels (x-axis). All relative risks are in reference to a river height increase of 6 m. (A—H) represent the response functions at lag weeks 1 through 8. We can see from (D—F) that declines in river height at lag weeks 4–6 are associated with increased risk of diarrheal disease. (TIF) [file pmed.1002688.s006.tif]

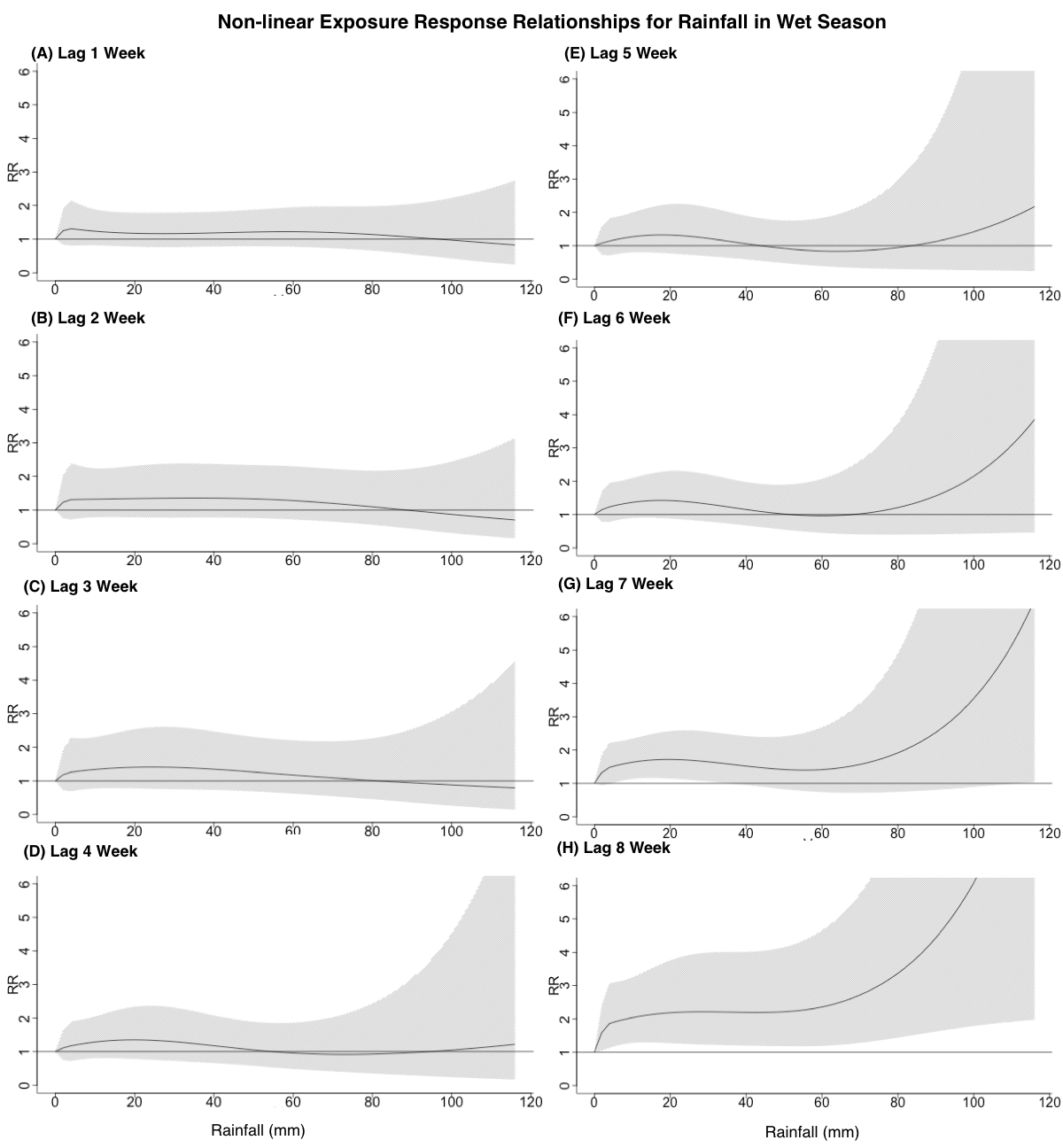

Supplement: S7 Fig — These relationships were generated using distributed lag nonlinear models that controlled for minimum temperature, maximum temperature, rainfall, and year. Relative risk estimates (black lines) and confidence intervals (grey areas) are shown across different rainfall levels (x-axis). All relative risks are in reference to rainfall of 0 mm. (A—H) represent the response functions at lag weeks 1 through 8. We can see from (G) and (H) that increases in rainfall at lag weeks 7 and 8 are associated with increased risk of diarrheal disease. (PNG) [file pmed.1002688.s007.png]
