# Supplementary material for: Hydrometeorology and flood pulse dynamics drive diarrheal disease outbreaks and increase vulnerability to climate change in surface-water-dependent populations: A retrospective analysis
Source: PLoS Med. 2018 Nov 8;15(11):e1002688. doi: 10.1371/journal.pmed.1002688 (PMC6224043; doi:10.1371/journal.pmed.1002688)
Supplement: S1 Text — (DOCX) [file pmed.1002688.s013.docx]

**Comparative Analysis with Distributed Lag Non-Linear Models**

We also used distributed lag non-linear models (DLNMs) to test our data. DLNMs are powerful models that allow for non-linear relationships between predictors and the outcome across space and time. They were initially developed to explore the lagged and non-linear relationships between one or two environmental predictors and a health outcome (e.g. the effects of temperature and air pollution on mortality).

We ran two DLNMs that included all environmental variables (maximum temperature, minimum temperature, rainfall, and river height) as predictors of diarrhea incidence. All predictors were lagged from 0 to 8 weeks. Non-linearity of the exposure-response relationships and lag structures were modeled using natural splines with 4 degrees of freedom. We used a quasipoisson model structure, because negative binominal regression is not supported within the DLNM structure.

In the dry season, we saw that decreasing river height was associated with a significantly higher risk of diarrheal disease at lag weeks 4-6 when controlling for all other variables and lags (Figure S6 below). In the wet season, higher rainfall was associated with larger diarrhea risk at lag weeks 7 and 8 (Figure S7 below). Associations between other environmental variables and diarrhea incidence were generally non-significant, which may be due to our moderate sample size. Indeed, we could not run DLNMs with water quality predictors due to the sparsity of the water quality measurements. Of note, the estimated exposure response relationships from the DLNMs are relatively linear. We do not see evidence of threshold effects or effect direction reversals.

There are two main limitations of using DLNMs. First, inference from these models, which have very high dimensionality, is limited by the sparsity of our water quality data and our sample size. In addition, DLNMs do not fit within the multimodel inference framework. Multimodel inference enables inference and prediction across many different models, precluding the choice of one “best” model. Many of the model specifications perform similarly well, and multimodel inference allows us to estimate the effects and importance of different predictors and lag times from many different models. Non-linear models are not supported within the multimodel inference framework, but as we showed with the DLNMs, the assumption of linearity is not unreasonable for this analysis.
